# Artificial Production of Bone

**Published:** 1859-08

**Authors:** 


### ARTIFICIAL PRODUCTION OF BONE.

M. Ollier, a young and extremely ingenious surgeon of Lyons, has lately published several important papers on the preservation of the periosteum in resection of bone, and has related most interesting experiments to show how large a share the periosteum takes in the regeneration of osseous matter. M. Ollier is pursuing his valuable labors, and has lately presented the Academy of Sciences, through M. Valpeau, some preparations illustrating certain points in the generation of bone by the periosteum. Amongst them was a portion of periosteum taken from the leg of a cock, which membrane, having been transplanted into the crest of the same bird, has given rise to a pretty considerable quantity of bone. The same result was obtained by introducing under the skin, and in the inguinal region of a rabbit, a portion of periosteum taken from the radius of the same animal. M. Ollier also transplanted a piece of periosteum of one rabbit upon another animal of the same kind, and likewise from a rabbit upon a dog; all which experiments were successful. —*Ib.*

